# Neuroprotective Effects of Musk of Muskrat on Transient Focal Cerebral Ischemia in Rats

**DOI:** 10.1155/2019/9817949

**Published:** 2019-06-25

**Authors:** Donghun Lee, Young-Sik Kim, Jungbin Song, Hocheol Kim

**Affiliations:** ^1^Department of Herbal Pharmacology, College of Korean Medicine, Gachon University, 1342 Seongnamdae-ro, Sujeong-gu, Seongnam-si, Gyeonggi-do 13120, Republic of Korea; ^2^Department of Herbal Pharmacology, College of Korean Medicine, Kyung Hee University, 26 Kyungheedae-ro, Dongdaemun-gu, Seoul 02447, Republic of Korea

## Abstract

Musk of musk deer has been one of the most precious traditional medicinal materials for treatment of stroke, but trading is prohibited. Musk of muskrat,* Ondatra zibethicus*, is an accessible substitute for musk of musk deer. However, neuroprotective effects of the musk of muskrat on stroke model are so far unclear. Aim of the study is to determine neuroprotective effects of the musk of muskrat on focal cerebral ischemia. The protective effects against focal cerebral ischemia were evaluated using a model of middle cerebral artery occlusion (90-minute occlusion followed by 24-hour reperfusion). Musk of muskrat was collected from scent bag of muskrat and orally administered at doses of 100 and 300 mg/kg twice at times of 0 and 90 min after occlusion. The effects on sensorimotor dysfunction were investigated by using balance beam test and rotarod test after brain ischemia. The expression of cyclooxygenase-2 (COX-2) was investigated by immunohistochemistry. Oral administration of musk at 300 mg/kg significantly reduced (*p*<0.001) the infarct volume by 32.4% compared with a vehicle-treated group. Oral administration of musk at 300 mg/kg also ameliorated ischemia-induced spontaneous and vestibule sensorimotor dysfunction in balance beam test and rotarod test compared with control group and COX-2 upregulation. Musk of muskrat may have neuroprotective effects against transient focal cerebral ischemia with recovery of sensorimotor dysfunction. Regarding the immunohistochemistry, the effects of muskrat may be due to anti-inflammatory properties through inhibition of COX-2 expressions.

## 1. Introduction

Musk is a collective name for a substance with a penetrating odor obtained from a gland of musk animals including African civet, sperm whale, or muskrat. But, in general, when it is called musk, it is known as secretions from preputial gland of the male musk deer. Musk of musk deer is essential component of Woohwangcheongsimwon as one of the representative medicinal materials for stroke treatment [1]. Its traditional use for stroke treatment has also been checked with focal ischemia animal model [2, 3]. However, there has been a growing need for an alternative of musk deer due to the restriction of its trade by Convention on International Trade in Endangered Species of Wild Fauna and Flora (CITES) since 1973.

Substances such as civet of African civet, ambergris of sperm whale, and musk of muskrat are known substitutes for musk of musk deer [4]. Among them, citvet was proved to be the main vector of severe acute respiratory syndrome (SARS), and as a result breeding citvet became impossible. Ambergris of cachalot is restricted item by CITES like musk of musk deer. On the other hand, musk of muskrat is the easiest securable alternative as muskrats are easy to breed and manage and very prolific [5].

The use of musk of muskrat, secretions of hypogastric scent bag of* Ondatra zibethicus*, has never been recorded in traditional medicinal references. In 1996, it was first recorded in Zhongguodongwuyaozhi that musk of muskrat could treat stroke, abscess, and swelling as it reduces inflammation, relieves pain, activates blood, and opens the orifices with aroma. It is also recorded in Zhongyaoxue that musk of muskrat could be used for both external and internal use as a substitute for musk of musk deer. Musk of muskrat consists of similar ingredients with musk of musk deer. It is known that musk of muskrat contains l-muscone, a key component, and macrocyclic musk compounds like civetone, cycloheptadecanone, cyclopentadecanone, cyclododecanone, and 22 kinds of C19-C26 fatty acids, sterol compounds, 19 kinds of esters, et cetera [6–8]. But Kim et al. reported that musk of muskrat contains cyclohexadecanone, which is a constitutional isomer of l-muscone, instead of l-muscone itself [5].

As musk of muskrat gets more interest for an alternative medicine for musk of musk deer, pharmacological effects are reported to possess anti-inflammatory, anticoagulant, analgesic, and hypotensive effects [5, 6, 9, 10], like musk of musk deer. However, there has been no report about whether it is effective on focal cerebral ischemia, which mimics ischemic stroke.

The aim of present study is to determine the neuroprotective effects of musk of muskrat on stroke animal model. To achieve this, we estimated the effect of musk of muskrat on brain infarct volume, sensorimotor dysfunction, and the expression of COX-2 involved in inflammation on middle cerebral artery occlusion (MCAo) rat model.

## 2. Materials and Methods

### 2.1. Sample

Musk of muskrat was bought from Muskland Co. (Jochiwon, Korea) and was kept in refrigerator as oil form for being used at this research. Muskland collected scent bag of muskrat,* Ondatra zibethicus*, and found that it contained 8.46% moisture, 87.0% crude fat, 0.01% ash, 0.024% total carbohydrate, and 1% protein.

### 2.2. Animals

Male Sprague-Dawley rats (300 ± 10 g) were obtained from Samtako Co. (Osan, Korea). Rats were housed under consistent temperature (23 ± 1°C) and humidity (55 ± 10%) on a 12-h light/dark cycle (light on at 07:00). Food and water were available* ad libitum*. The experiments were carried out in accordance with the Principle of Laboratory Animal Care (NIH Publication #85-23, revised 1985) and Kyung Hee University's Institutional Animal Care and Use Committee.

### 2.3. Surgery

Focal cerebral ischemia was induced by transient MCAo [11]. Briefly, rats were anesthetized under 2% isoflurane in a mixture of N_2_O/O_2_ (7:3) throughout the surgery. Left branch of carotid artery was exposed through a midline incision. The external carotid artery (ECA) was ligated and cut near the junction of the proximal ECA junction. The common carotid artery (CCA) and internal carotid artery (ICA) were temporarily blocked by vascular clips. A 4-0 nylon monofilament (diameter 370 ± 5 *μ*m) with a round silicone head was inserted into the ECA. Exact location of the suture was determined when the suture was inserted at a minimum of 18 mm from the CCA/ ICA junction. After 90 minutes of MCAo, the suture was removed to allow reperfusion. Rats in the sham operated group received the same surgical procedure except for a probe insertion. The rectal temperature was maintained at 37 ± 0.5°C until 6 hours after ischemia with a heating lamp and blanket system (Harvard Apparatus, Holliston, MA, USA). Occlusion of the MCA was confirmed by the presence of characteristic behavioural deficits, such as paralyzed forelimb flexion, torso twist, and spontaneous circling after reperfusion. Rats that failed to meet these criteria were excluded from the study.

### 2.4. Sample Treatment

Musk of muskrat was dissolved in aqueous solution of tween 20 (5%, w/v) and administered orally twice at doses of 100 and 300 mg/kg at 0 and 90 min after occlusion. The rats in the vehicle-treated group were given aqueous solution of tween 20 (5%, w/v). Treatment was blinded.

### 2.5. Balance Beam Test

The balance beam test was performed at 22 hours after ischemia by modifying the previously described [12]. The rats were placed in the middle of a wooden square bar (width 2.5 cm, length 122 cm, and height 42 cm) and scored as follows: 0 = the rat was not able to stay on the beam; 1 = the rat did not move, but was able to stay on the beam; 2 = the rat tried to traverse the beam, but fell; 3 = the rat traversed the beam with more than 50% footslips of the affected hindlimb; 4 = the rat traversed the beam with more than one footslip, but less than 50%; 5 = the rat had only one slip of the hindlimb; and 6 = the rat traversed the beam without any slips of the hindlimb.

### 2.6. Rotarod Test

The rotarod test was performed at 22 hours after ischemia. Rats were placed onto an accelerating rotarod (from 0 to 40 rpm; Ugo Basile, Milan, Italy) and the time from when the rats fell of the rotarod was measured. For each rat, latency times were recorded in five separate trials. The highest and lowest values were excluded and the mean of the remaining three trial results was used for the analysis.

### 2.7. Tissue Preparation

Twenty-four hours after MCAo, the rats were anesthetized and decapitated. For measuring infarct volume, the decapitated rat brain was carefully removed and cut into 6 coronal sections of 2 mm thickness. The sections were stained with 2% TTC (2,3,5-triphenyltetrazolium chloride; Sigma, USA) in saline at 37°C for 30 minutes. Immunohistochemical staining was performed by perfusion with 4% paraformaldehyde after 24 hours of ischemia and heparinized 5% sodium nitrite saline solution. The brain was removed and cut into 4 *μ*m sections using a cryocut (3050s; Leica, Germany).

### 2.8. Measurement of Infarct Volume

TTC-stained sections were analysed for infarct volume using a computerized image analysis system (Image ProPlus, Media Cybernetics, USA). Correlated infarct volume (mm3) was calculated from the total volume of the contralateral hemisphere minus the unimpaired volume of the ipsilateral hemisphere. The infarct volume (%) was calculated by dividing the correlated infarct volume with the total volume of the opposite hemisphere.

### 2.9. Immunohistochemistry

Immunohistochemistry was performed by modifying the previously described [13]. Brains were removed, fixed, and cut into 40-*μ*m sections using a cryostat (Cell Signaling, USA). Free-floating sections were reacted with a rabbit polyclonal antibody against COX-2 (1:100; Abcam, UK) overnight at room temperature. Subsequently, the sections were reacted with biotinylated rabbit antibody (1:200; Sigma Aldrich, USA) and incubated with avidin-biotin complex reagent (Vector Laboratories, USA) for 1 h. The sections were visualised with 0.05% 3,3-diaminobenzidine solution (Sigma Aldrich) containing hydrogen peroxide.

### 2.10. Statistics

Statistical difference between three groups was analyzed using one-way analysis of variance (ANOVA) followed by Dunnett's post hoc test. Difference between two groups was analysed using independent t-test (GraphPad Prism 5.0, GraphPad Software, USA). Statistical significance was accepted at p<0.05 in Dunnett's test. Data were expressed as mean ± standard error of the mean.

## 3. Results

### 3.1. Effects on Infarct Volume

**Figure 1:**
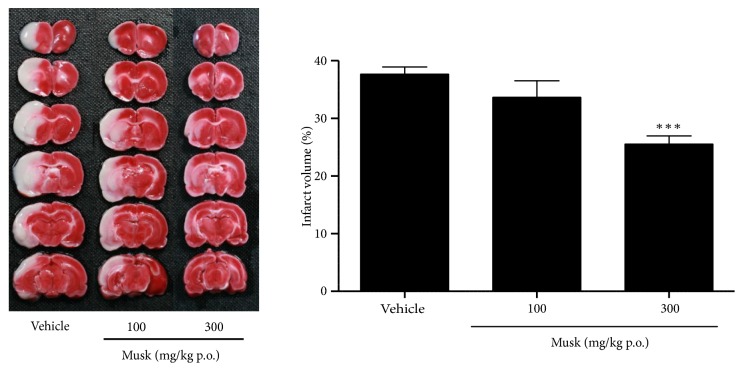
Dose-dependent effect of musk of muskrat on infarct volume induced by MCAo. N=12 per group; *∗∗∗*p<0.001 vs. Vehicle-treated control by one-way ANOVA with* post hoc* Dunnett's test.

To determine the neuroprotective effect of musk of muskrat, coronal sections were obtained after 24 h of induction. The white area indicates the infarct area in the bottom (Figure 1). It extended from the caudoputamen, parietal cortex, and temporal cortex to the penumbral region after MCAo. The vehicle-treated group showed 36.69±1.42% of infarct volume, while musk-treated group showed 33.37±2.93% and 25.18±1.64% at 100 and 300 mg/kg, respectively. Oral administration of musk of muskrat at 300 mg/kg significantly reduced the infarct volume by 32.4% compared with vehicle-treated group, respectively. Oral administration of musk of muskrat at 100 mg/kg showed moderate tendency to decrease but there was no significant difference because of its high deviation.

### 3.2. Effects on Balance Beam and Rotarod Tests

To see whether the protective effects of musk of muskrat associate with any functional recovery, we investigated balance beam test and rotarod test which are commonly used to determine the ameliorating effect on motor coordination, sensory motor integration, and spontaneous locomotion.

**Figure 2:**
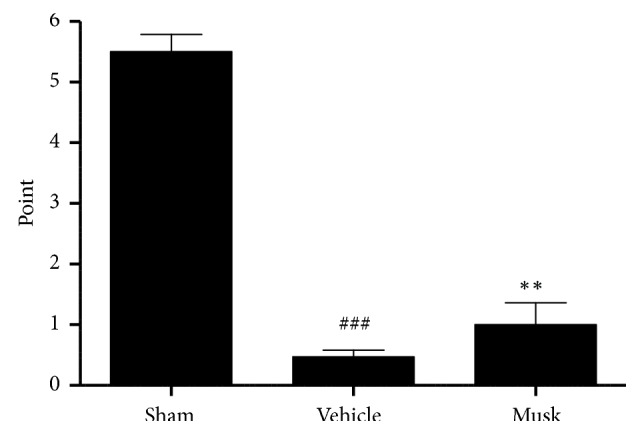
The effect of musk of muskrat on balance beam test after MCAo. Musk; oral administration of musk of muskrat at dose of 300 mg/kg. N=5 per group; ### p < 0.001 vs. Sham group, *∗∗* p < 0.01 vs. vehicle-treated group.

**Figure 3:**
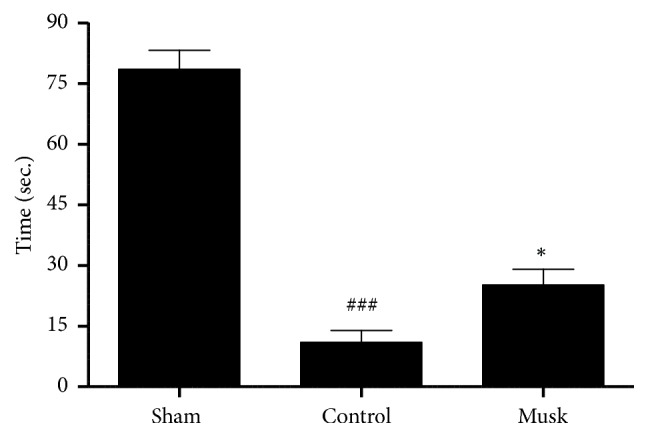
The effect of musk of muskrat on rotarod test after MCAo. Musk; oral administration of musk of muskrat at dose of 300 mg/kg. N=5 per group; ### p < 0.001 vs. Sham group, *∗* p < 0.05 vs. vehicle-treated group.

Rats in the vehicle-treated group scored significantly lower on the balance beam test than did those in the sham-operated group (0.5 ± 0.1 vs. 5.5 ± 0.3 points; p<0.001); however, rats that received 300 mg/kg musk of muskrat scored higher than those in the vehicle-treated group (1.0 ± 0.4 points vs. 0.5 ± 0.1 points; p < 0.01; Figure 2). In the rotarod test, vehicle-treated group significantly decreased compared with sham-operated group (11.0 ± 2.9 s vs. 78.5 ± 4.7 s); however, rats received 300 mg/kg of musk of muskrat significantly prolonged the latency time to 25.2 ± 3.9 s (p < 0.05; Figure 3).

### 3.3. Effects on COX-2 Expression

**Figure 4:**
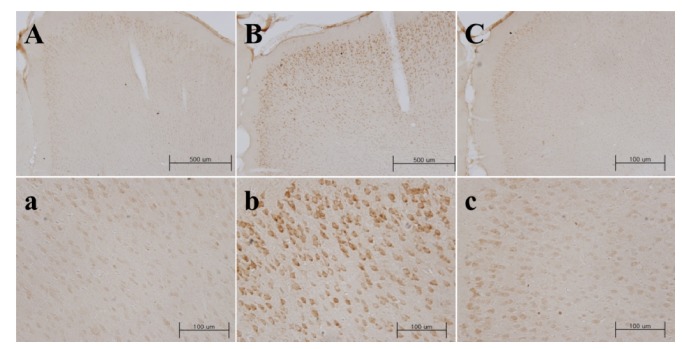
Inhibitory effect of musk of muskrat on COX-2 expressions (A, B, C) in the peri-infarct cortex, 24 hours after 90 minutes of MCAo. Sham (A, a), vehicle administered group (B, b), musk of musk rat administered group (300 mg/kg, p.o.; C, c). Boxed regions in A, B, and C (x40) are shown in a, b, and c (x400), respectively.

To define early change in COX-2, immunohistochemistry was performed at 24 hours after induction. COX-2 expressions in the peri-infarct cortex were increased in vehicle-administered group compared with sham group. In the group treated with musk of muskrat, COX-2 expression was remarkably decreased and restricted to the core part of the ipsilateral hemisphere. The peripheral part was hardly stained (Figure 4).

## 4. Discussions

Oral administration of musk of muskrat at doses of 300 mg/kg at 0 min and 90 min after MCAo reduced the infarct volume significantly and ameliorated spontaneous and vestibule sensorimotor dysfunction in balance beam test and rotarod test compared with control group, respectively. Also it was found that it restrained COX-2 expression markedly when measuring after 24 h.

MCAo model is known as the most suitable model for stroke treatment research because it induces focal cerebral ischemia by occluding proximal of middle cerebral artery where ischemic stroke occurs most frequently [14, 15]. Because there is the coexistence of necrosis of ischemic core and apoptosis spreading to penumbra region in MCAo model, it is the most analogous model with clinical stoke patients' pathophysiological and behavior pattern including extracellular edema and blood circulatory system intervention [16]. Both in clinical stroke and in MCAo model, necrosis at ischemic core cannot be protected without reperfusion therapy within 3 h; nevertheless it is known to be almost clinically unfeasible [16]. Meanwhile, in apoptosis arising in penumbra region neurons, death of neurons can be inhibited by neuroprotective substances [17, 18]. The brain infarct volume is the most important index to confirm the medicinal effects on injury of necrosis or apoptosis from focal ischemic stroke induced by MCAo model [19] and estimation of 90 min of MCAo-induced brain infarct volume after 24 h by TTC staining is known to be one of the most suitable conditions for evaluating the effects of sample due to its clear induction and low variation [20]. In this study, oral administration of musk of muskrat at doses of 300 mg/kg at 0 min and 90 min after MCAo significantly reduced brain infarct volume and infarct area was mostly restricted to ischemic core region. This result suggests that musk of muskrat can inhibit neuronal damage at penumbra region, which means it could be neuroprotective substance at focal cerebral ischemia.

To define whether neuroprotective effects of musk of muskrat associate with protective effects on sensorimotor dysfunction from brain damage, balance beam test and rotarod test were conducted. Brain injury is a form of physical impairment, accompanied by sensorimotor dysfunction [21], and whether the sample ameliorates sensorimotor dysfunction is important to determine whether to conduct clinical trials [22]. The balance beam and rotarod tests are both commonly used to assess motor coordination and balance alterations following MCAo [12, 23]. These tests are also known to have considerable correlation with evaluation of locomotion by MCAo brain damage [24]. In this study, the reduction in infarct volume was accompanied by elevated balance beam score and prolonged rotarod latency after musk of muskrat treatment. The results suggest that the protective effect of musk of muskrat in cerebral cortex and corpus striatum injury is associated with a restoration of the ischemia-induced sensorimotor dysfunction, suggesting that musk of muskrat could help functional restoration after ischemia.

Herein, musk of muskrat inhibited COX-2 upregulation induced by MCAo in ipsilateral neocortex. Focal cerebral ischemia triggers an inflammatory reaction, which is known as the main factor to accelerate brain damage [25]. After several hours from ischemic stroke, blood brain barrier is collapsed and leukocytes invade in large scale in succession and mass production of inflammatory cytokine accelerates tissue damage, brain edema, and glial activation [26]. COX-2 is rate-limiting enzyme in charge of inflammatory reaction by transforming arachidonic acid into prostaglandin endoperoxide H2 [27]. COX-2 expression increases as of the activation of NMDA receptor from excessive glutamate release [28] and the production of inflammatory cytokine in focal cerebral ischemia [29]. It is known that this increase of COX-2 expression is one of the main reasons of secondary damage at ischemic stroke and, also, it has proved that selective COX-2 inhibitor or COX-2 gene deletion shows neuroprotective effect [30–33]. These results suggest that neuroprotective effects of musk of muskrat after focal cerebral ischemia might be attributable to interrupting inflammatory reaction by the inhibition of COX-2 expression.

## 5. Conclusion

Musk of muskrat protects neurons against focal cerebral ischemia in rats with functional restoration. In relation to the immunohistochemical studies, the effects of musk of muskrat may be due to their anti-inflammatory properties by inhibiting COX-2 expression. Based on these findings, it is tempting to suppose that musk of muskrat could be considered as a substitute for musk of musk deer in view of the traditional use of stroke treatment.

## Data Availability

The experimental data used to support the findings of this study are available from the corresponding author upon request.
